# A process evaluation of systematic risk and needs assessment for caregivers in specialised palliative care

**DOI:** 10.1186/s12904-017-0196-x

**Published:** 2017-04-08

**Authors:** Kia Toft Thomsen, Mai-Britt Guldin, Mette Kjærgaard Nielsen, Chaitali Laura Ollars, Anders Bonde Jensen

**Affiliations:** 1 Department of Oncology, Aarhus University Hospital, Noerrebrogade 44, Aarhus C, 8000 Denmark; 2 Research Unit for General Practice, Department of Public Health, Aarhus University, Bartholins Allé 2, Aarhus C, 8000 Denmark; 3 The Palliative Team, Department of Oncology, Aarhus University Hospital, Noerrebrogade 44, Aarhus C, 8000 Denmark

**Keywords:** Family caregiver, Palliative care, Caregiver support, Needs assessment, Bereavement risk assessment

## Abstract

**Background:**

Caregiving is strenuous and it may be associated with adverse psychological outcomes. During the palliative care trajectory, there are unique opportunities for providing support and preventing poor bereavement outcome. However, the tasks of palliative care staff in relation to caregivers are often unclear in the daily practice. Assessment is recommended to establish risk and needs and standards for caregiver support are available. Still, the feasibility of applying these standards among caregivers in everyday clinical practice has not been tested so far.

**Methods:**

This study tested the feasibility of an intervention based on key elements of the “Bereavement support standards for specialist palliative care services” in a Danish specialised palliative home care team. We followed the UK Medical Research Council’s guidelines for the process evaluation of complex interventions. The intervention consisted of: 1. Systematic risk and needs assessment for caregivers at care entry; 2. Interdisciplinary conference to prepare a support plan; 3. Targeted support; 4. The establishment of an electronic medical record for caregivers to document targeted support. Outcomes included the reach, fidelity and acceptability of the intervention as well as the assessment of contextual factors.

**Results:**

The intervention reached 76 of 164 caregivers (46%). The interdisciplinary risk assessment and documentation of a support plan was conducted in 57 (75%) of the enrolled caregivers. Finally, a separate medical record was established according to the intervention blueprint for 62% of caregivers receiving targeted support. After managing initial challenges, palliative care staff reported that the intervention was useful and acceptable.

**Conclusion:**

The intervention proved feasible and useful. Still, we identified barriers to the implementation which should be taken into consideration when planning implementation of a systematic risk and needs assessment and in the establishment of medical records for caregivers.

## Background

Caregivers constitute an important source of support for patients in palliative care. However, caregiving is often strenuous and may be associated with adverse psychological outcomes [1–4]. Thus, caregivers may need support to fulfill their task. Several studies have tested interventions aiming at reducing the distress associated with caregiving [5–9]. Still, support should be delivered according to the individual caregiver’s need [10, 11]. During the palliative care trajectory, there are unique opportunities for assessing the need for support and risk of poor bereavement outcomes among caregivers. Although support to caregivers during illness and after the patient’s death is defined as part of palliative care [12], it is often unclear which procedures and routines should be adopted by palliative care staff in daily clinical practice [13]. A recent survey by the EAPC Bereavement Care Taskforce showed that only a quarter of 302 European palliative care services used a formal risk assessment tool, and that 197 (66%) services stated that their bereavement support was not based on any formal policy or guideline [14]. Until recently, no validated needs assessment tool for caregivers in palliative care existed [15]. Without formalised risk and needs assessment, support to caregivers in palliative care may be coincidental, and it is unclear whether support reaches those in need [10, 16]. Findings from a Danish study underlined these problems showing that an ad hoc risk assessment conducted by specialised palliative professionals only correctly predicted about 20% of the bereaved individuals who developed complicated grief. Standards to ensure high quality in caregiver support have been developed in the *“Bereavement support standards for specialist palliative care services (“Bereavement support standards”)* [11]. These standards are based on a review of international evidence as well as recommendations from expert advisory groups [11]. The standards suggest structured caregiver risk assessment at care entry, review in interdisciplinary team meetings and structured documentation of assessments [11]. Previous studies have investigated measures to assess caregivers’ needs and risk of adverse bereavement outcomes [17–19]. No prior studies have tested the feasibility of a systematic discussion of caregivers’ risks and needs at interdisciplinary conferences. One study described the use of separate medical records for caregivers, but it did not assess the feasibility of this procedure [13]. To our knowledge, the feasibility of implementing the “*Bereavement support standards”* in everyday clinical practice has not yet been tested. Choosing the appropriate risk assessment methods as well as deciding where and how to document assessments represents some of the challenges for applying the standards in a clinical setting. The aim of this study was to evaluate the feasibility of an intervention for caregivers based on key elements of the “*Bereavement support standards*” according to the UK Medical Research Council’s guidelines for the process evaluation of complex interventions [20]. In this study, a *risk and needs assessment form* was developed, based on the risk factors listed in the *“Bereavement support standards”,* and the establishment of an electronic medical record for caregivers was made possible.

## Methods

### Setting

The study was conducted in the framework of the palliative home care team (PCT) at Aarhus University Hospital, Denmark, an outpatient unit which provides specialised palliative services to a population of 366,000 inhabitants. The PCT staff consisted of five doctors, five nurses, one physiotherapist, one psychologist, one chaplain, one social worker and two secretaries. In 2015, 483 patients were referred and 372 received services from the PCT. The majority (94%) of patients had cancer. The average time from the first home visit to death of the patient was 63 days. The “usual care” caregiver support consisted of an informal assessment of risks and needs at not predefined time points during the end-of-life caregiving trajectory. Caregivers’ needs and risk were discussed at the interdisciplinary conference when needed, though not systematically. Based on an ad hoc clinical assessment of caregivers’ needs, caregivers could be offered support from the interdisciplinary team, e.g. support from a nurse, chaplain or psychologist.

### Study design

The study was designed as a one group prospective caregiver intervention. The research population was the PCT staff (*n* = 16). Caregivers were enrolled between 1 March and 31 December 2015. All eligible caregivers to newly referred patients present at the first visit by the PCT in the patient’s home were invited to participate in the study. Inclusion criteria were (1) age > 18 years and (2) willing to give written informed consent. PCT staff excluded caregivers with cognitive and/or mental impairment and caregivers in a situation where clinical issues, e.g. if the patient was actively dying or in great pain, obviously prevented participation.

### Intervention

The “*Bereavement support standards”* [11] were chosen as a framework for the intervention. An intervention consisting of four components was designed: 1) At the first home visit, caregivers filled in a *risk and needs assessment form*; 2) at the subsequent interdisciplinary conference the caregiver’s risk factors and support needs were discussed based on the completed form, and a support plan was documented in the patient’s medical record. 3) On the basis of the assessment, targeted support from the interdisciplinary team could be offered to caregivers. Support options included a formalised supportive caregiver session with a nurse, a psychologist (in order to assess the need for psychotherapeutic support), a chaplain, volunteer support or referral to the general practitioner to be assessed for e.g. depression 4) Finally, technical and legal steps were taken to enable the establishment of a separate electronic medical record for caregivers to document the targeted support they received.

### Risk and needs assessment form

Since no standardised risk screening tool for caregivers was recommended by the “*Bereavement support standards*” and no validated assessment tool was available in Danish, an assessment form was developed based on the risk factors listed in the “*bereavement support standards*” and an existing literature review on risk factors for adverse bereavement outcome [11, 21]. The first draft was evaluated at a project meeting with the PCT staff and pilot tested among ten caregivers to patients receiving care by the PCT. The form was further revised in a continuous process at project meetings with the PCT staff. During this revision, it became clear that the form did not assess current needs, which seemed strange to the PCT staff in the contact with caregivers. Thus, the form was elaborated to reflect caregiver’s current support needs with inspiration from the development of the Carer Support Needs Assessment Tool (CSNAT) [17, 22]. PCT staff reported that two items were too confrontational: “Are you worried about how to get by if your relative dies from his/her disease?” and “Is it difficult to talk about the fact that your relative’s disease is a serious disease that one can die from?” These items were replaced with items that focused on distress in the caregiver’s current situation, e.g. “Do you need help to cope with your own thoughts, feelings or worries?” and “Do you need knowledge about what to expect in the course of your relative’s illness?” The *risk and needs assessment form* was finalized as a 17-item questionnaire, depicted in Table 1. Caregivers could indicate the degree to which they agreed to the statement in each item in four response categories (*not at all, a little, some, a lot)*. PCT staff could ask caregivers to elaborate on items indicating high need or high risk. The assessment form was intended to guide health care professionals to have a subsequent exploratory dialogue with the caregivers in accordance with the “*Bereavement support standards*” [11].

**Table 1:** Items of the risk and needs assessment form for caregivers^a^

What is your relation to you ill relative?				
Do you have children in your household that you are (partly) responsible for?				
	Not at all	A little	Some	A lot
Do you need information about your relative’s disease and/or your current situation?	⎕	⎕	⎕	⎕
Do you and your relative need further help to alleviate disease symptoms, including giving medicine?	⎕	⎕	⎕	⎕
Do you need information about who to contact if you need help, including at night?	⎕	⎕	⎕	⎕
Do you need help to clarify economic, legal, housing or work-related issues?	⎕	⎕	⎕	⎕
Do you need knowledge about what to expect in the course of your relative’s disease?	⎕	⎕	⎕	⎕
Do you need support to talk with your relative about his/her disease and its consequences?	⎕	⎕	⎕	⎕
Do you feel as if you lack support from family and friends?	⎕	⎕	⎕	⎕
Do you feel overwhelmed by practical tasks or tasks in relation to caring for your ill relative?	⎕	⎕	⎕	⎕
Do you need help to take breaks from caring for your ill relative, or taking care of your own needs?	⎕	⎕	⎕	⎕
Do you need help to cope with your own thoughts, feelings or worries?	⎕	⎕	⎕	⎕
Do you have thoughts about religion/spirituality or life and death, that you feel you need to talk about?	⎕	⎕	⎕	⎕
Do you feel depressed?	⎕	⎕	⎕	⎕
Have you experienced significant losses earlier in your life that places a strain on you now? (e.g. regarding health, job, divorce, or death?)	⎕	⎕	⎕	⎕
Do you suffer from a mental illness (e.g. depression, stress or anxiety) that has been diagnosed by a medical doctor?	Yes ⎕		No ⎕	
If yes, which illness?				

^a^The Danish risk and needs assessment form was translated from Danish into English for the purpose of this article

### The medical research council framework

Evaluating the feasibility of the intervention was done according to the UK Medical Research Council’s (MRC) framework for the process evaluation of complex interventions [20]. The framework recommends a clear description of the intervention and how it is expected to work, ideally depicted in a logic model [7, 9] (Table 2). The focus of a process evaluation varies according to the stage at which it is conducted [20]. Our focus was the feasibility of the intervention; we chose to include: 1. Description of the intervention and its causal assumptions, 2. The implementation process: What was delivered and how? and 3. The interaction between the intervention and the context: any external factors in the intervention that may have acted as a barrier or facilitator to its implementation [20].

**Table 2:** Overview of the intervention in a logic model^a^

Intervention inputs	Intervention components	Feasibility outcomes	Outcomes for caregiver support procedures	Impact
Bereavement support standards for caregivers in palliative care^b^ Risk and needs assessment form based on standards^b^ and systematic literature review^c^ Possibility of creating electronic medical record for caregivers	1. Systematic risk and needs assessment at care entry 2. Interdisciplinary conference to prepare support plan 3. Targeted support from interdisciplinary team 4. Establishment of an electronic medical record for caregivers to document targeted support	Reach, fidelity and acceptability of intervention components Contextual factors: Any external factors to the intervention that may act as a barrier or facilitator to its implementation	1. Ensures focus on caregivers, exposes needs swiftly, allows targeted support initiation 2. Ensures interdisciplinary assessment of risks and needs 3. Support is tailored to caregivers’ needs 4. Enables systematic planning of intervention and sharing of information between professionals, secures follow up	Reduces distress during caregiving and prevents distress following bereavement Targeted support: Support aligns with needs Improved use of resources in palliative care

^a^Logic Model according to W.K. Kellogg Foundation [23]

^b^Bereavement support standards for specialist palliative care services, Hall et al. 2012 [11]

^c^Lobb et al. 2010 [21]

### Process evaluation outcomes

The outcomes of the process evaluation were: Reach (whether the intended audience came into contact with the intervention), fidelity, acceptability and contextual factors [7, 10]. *Reach* was assessed through participation rate and the number of caregivers offered targeted support (with nurse or psychologist). *Fidelity* was assessed by the number of interdisciplinary conferences resulting in a support plan for the caregiver documented in the medical record, and the number of separate medical records established for caregivers receiving targeted support from the PCT staff. Fidelity outcomes were assessed by examining the medical records of patients and caregivers. *Acceptability* was investigated through continuous unstructured group interviews with PCT staff during the implementation period. Furthermore, a survey was conducted among the doctors and nurses in the PCT (*n* = 9) who introduced the *risk and needs assessment form* to caregivers, to examine the acceptability of the assessment form itself. *Contextual factors* were assessed by considering factors in the context acting as facilitators or barriers to the implementation. We used descriptive statistics to summarize the data, including simple counts and percentages.

### Ethics, consent and permissions

All participants were informed about the purpose of the study, that participation was voluntary and that they could withdraw at any time. It was explained that the data would be anonymized. All participants gave written informed consent to participate. The research protocol was submitted for approval by The Central Denmark Region Committees on Health Research Ethics but no ethics approval was needed to conduct this study (reference number 211/2013). Approval was obtained from the Danish Health Authority (reference number 1-16-02-456-15).

## Results

Table 3 provides an overview of the barriers and process evaluation results for each of the four intervention components.

**Table 3:** Overview of process evaluation results

Intervention components	Feasibility outcomes	Barriers to implementation
1. Systematic risk and needs assessment at care entry	• Participation rate was 46%. • Systematic risk and needs assessment was conducted for all included caregivers at enrollment (*n* = 76).	• Ad hoc assessment was considered sufficient by palliative care staff, resulting in reservations in conducting systematic assessment. • Shortage of time at the first home visit to conduct caregiver assessment. • Caution with confronting caregivers with severity of disease at first home visit and eliciting strong emotional responses.
2. Interdisciplinary conference to prepare support plan	• Interdisciplinary risk and needs assessment and support plan was conducted for 75% of caregivers.	• Time shortage at conference • Concern that focus is directed away from patient.
3. Targeted support from interdisciplinary team	• Targeted support was offered to 29% of caregivers.	• Indistinctness in professional role distribution.
4. Establishment of electronic medical record for caregivers to document targeted support	• A medical record was established in accordance with the intervention blueprint in 62% of caregivers who received targeted support	• Ethical considerations about medical records for caregivers with no formal diagnosis. • Reservation regarding sharing information with emotional content.

### Reach

Overall, 164 family caregivers were assessed for eligibility during the inclusion period. In total, 119 caregivers (73%) met inclusion criteria and agreed to receive information about the study. Of these, 76 (64%) returned written informed consent and completed the *risk and needs assessment form*. Overall, the intervention reached 76 of the 164 caregivers (46%) included in the study. The main reason for not asking caregivers to participate was that the PCT staff deemed it inappropriate, e.g. “the patient was dying when we arrived” or “we admitted the patient to the hospital right away.” Targeted support was offered to 22 (29%) of the 76 included caregivers. However, the targeted support was only carried out in 13 cases (17% of included caregivers). In the remaining nine cases, the caregiver either declined, or the patient died or was terminated from PCT services before the targeted support was initiated.

### Fidelity

Caregiver risk and needs assessment was discussed at the interdisciplinary conference and a support plan was documented in 57 (75%) of the included caregivers (*n* = 76). In ten cases (13%), no interdisciplinary conference was held, either because the patient died or was transferred to hospice shortly after referral to the PCT. In nine (12%) cases, the support plan focused on the patient, e.g. symptom management or place of care, and a support plan for the caregiver was not documented. The procedure for establishing an electronic medical record was followed for eight out of the 13 caregivers (62%) who received targeted support. Five medical records were established in the caregiver’s name, and in three cases, the psychologist had a session with the patient and the caregiver together, which was documented in the patient’s medical record. In the remaining five cases (38%) a separate medical record should have been established according to the intervention blueprint, but this did not happen.

### Acceptability

In the beginning of the implementation period, PCT staff discussed whether systematic risk and needs assessment was necessary; they felt that the “usual care,” i.e. ad hoc risk and needs assessment, was sufficient. In the unstructured group interviews, PCT staff reported a reluctance to introduce the *risk and needs assessment form*, because completing it elicited emotional responses in some caregivers, which the staff felt they did not have time to take proper care of at the first home visit. PCT staff also reported a shortage of time at the interdisciplinary conference. As each referred patient had multiple complex problems, there was a concern among the staff that focus was directed from the patient to the caregiver. Further, there was a lack of distinctness in professional role distribution when deciding what type of support (i.e. nurse, psychologist, pastoral care), should be offered to caregivers, as no guidelines for deciding this existed.

In the survey investigating the acceptability of the *risk and needs assessment form* at the end of the study period, all nurses and physicians (*n* = 9) agreed to the statements “the assessment form provides an overview of the caregiver’s needs” and “it gives me an idea about what to ask the caregiver when I see him/her again” (Table 4). A lack of time to introduce the *risk and needs assessment form* at the first home visit was reported by 6 (67%) nurses and physicians. During the implementation period, PCT staff generated their own pragmatic strategy for home visits with significant time constraints. They asked the caregiver to fill in the *risk and needs assessment form* whenever he or she could find the time and subsequently mail it to the PCT. In the unstructured group interviews, PCT staff stated that their overall effort to support caregivers early in the caregiving trajectory had intensified as a result of the intervention. Hence, the staff experienced that they used more resources in the caregiving period and consequently less resources in the bereavement period. The staff reported that the continuous contact during caregiving facilitated delivery of a more cohesive and preventive support compared to the sporadic contact after the patient’s death.

**Table 4:** Acceptability survey among physicians and nurses in the palliative care team (*n* = 9)

	Agree or strongly agree N (%)	Neither agree nor disagree N (%)	Disagree or strongly disagree N (%)
The assessment tool provides an overview of the caregiver’s needs	9 (100)	0 (0)	0 (0)
It gives me an idea about what to ask the caregiver when I see him/her again	9 (100)	0 (0)	0 (0)
The assessment tool gives me an idea of the caregiver’s risk of distress after the patient’s death	8 (89)	1 (11)	0 (0)
The assessment tool is helpful in the interdisciplinary work	7 (78)	2 (22)	0 (0)
It can be difficult to find time to introduce the assessment tool at the first home visit	6 (67)	3 (33)	0 (0)
The assessment tool does not provide the information I need to support the caregiver	0 (0)	0 (0)	9 (100)
It can be difficult to introduce the caregiver to the assessment tool	4 (44)	3 (33)	2 (22)
I get a sufficient impression of caregiver needs by talking informally to the caregiver and making my own observations, rather than using the assessment tool.	0 (0)	4 (44)	5 (56)

A thorough report of how caregivers experienced the intervention is beyond the scope of this study. However, reports from the PCT staff in the unstructured group interviews indicated that the systematic screening and targeted support was generally received well among caregivers. Furthermore, it was the impression that our *risk and needs assessment form* indeed captured the needs of the caregivers.

Establishing a medical record for the caregivers receiving targeted support from the PCT turned out to be controversial among the professionals. It was discussed whether it was ethically sound to create a medical record for people who did not have a formal diagnosis. PCT staff also expressed reservations about documenting information with emotional content in the electronic medical record, which was accessible for other health care professionals.

### Contextual factors

The main focus in palliative care is to manage patients’ symptoms. Hence, the clinical routines in palliative care have traditionally been set up to ensure assessment and alleviation of patients’ symptoms. The existing routines may have acted both as a barrier and a facilitator to the implementation. The caregiver intervention was adapted to fit into the existing well-known routines to the PCT staff, which may have facilitated the implementation. On the other hand, the intervention required an extension of focus to make a systematic assessment of caregivers’ risk and needs. The modification of existing procedures and attitudes among busy PCT staff is likely to have been a barrier to the implementation.

## Discussion

### Main findings

Systematic risk and needs assessment resulted in an individual support plan for 75% of participating caregivers, enabling structured follow-up and targeted support for this group. Overall, we argue that this intervention based on key elements of the *“Bereavement support standards*” was feasible. PCT staff reported some initial challenges but after a few adjustments, the intervention turned out to be both useful for and acceptable to the PCT staff. These results can potentially help ensure that the support in palliative care reaches the caregivers who need it.

### Systematic risk and needs assessment

A participation rate of 46% (76/164) is comparable to other studies in end-of-life care settings [5, 11] and we therefore consider the reach of the intervention satisfactory. An Australian population based study found that 35% of bereaved were at moderate risk for developing complicated grief, and a prior study conducted in 2006 in the same palliative home care team as the present study, found the prevalence of complicated grief to be 28% 13 months after the patient’s death [4]. These results correspond to the 29% who were offered targeted support based on the systematic risk and needs assessment in our study.

The interdisciplinary conference resulted in documented support plans for 75% (57/76) of the caregivers. To the best of our knowledge, no previous studies have tested the feasibility of systematically assessing caregivers’ needs and risk on interdisciplinary conferences. In nine cases (12%), the care plan focused on the patient only and did not mention the caregiver. Our data could not show if these nine caregivers were omitted from the interdisciplinary discussion, or if the discussion resulted in the decision that no support was necessary at the time resulting in no documentation of a support plan. This caused uncertainty afterwards about whether caregivers’ needs and risk had in fact been assessed. This points to the importance of documenting outcomes of the interdisciplinary conference in all cases. Such an adaptation may also improve the consistency of the risk and needs assessment and enable subsequent quality assessment.

In the beginning of the implementation period PCT staff felt that the ad hoc risk assessment they usually conducted was sufficient, which resulted in reservation against implementing the systematic risk and needs assessment. The staff also reported a reservation regarding asking the caregivers questions that could elicit emotional reactions. Similar challenges have been reported in other studies on systematic risk and needs assessment [24, 25]. An Australian study found a preference among staff for an informal chat rather than a formal risk assessment, and a concern about confronting caregivers with personal questions [24]. A British study on the implementation of the CSNAT needs assessment tool showed initial concern among staff members. Some felt that needs were best discussed as they arose, rather than presented “all in one go”, since it might bring up issues that the caregiver was not prepared to discuss [25]. In our study, the very same concerns resulted in a change of two items in the *risk and needs assessment form*, because they were deemed too confrontational.

However, during the implementation period there was a shift in attitudes among PCT staff. The staff recognized that the systematic risk and needs assessment generated relevant information that was not previously available to them. A new routine emerged where contact nurses kept the completed assessment form on their desk at the follow-up phone call after the first home visit, asking the caregiver to elaborate on items with a high score. This example of creative interaction with the intervention components showed that PCT staff took the intervention to heart and made the necessary adaptations to make it work in their everyday practice. PCT staff stated that their overall effort to support caregivers early in the caregiving trajectory intensified as a result of the intervention, and that the continuous contact during caregiving facilitated delivery of a more cohesive support compared to the sporadic contact after the patient’s death.

### Establishment of an electronic medical record for caregivers

The procedure for establishing an electronic medical record for caregivers was only followed for eight (62%) caregivers. The reason for the low fidelity for this intervention component is unknown. Caregivers’ civil registration number was a prerequisite for establishing the medical record. One possible explanation is that the civil registration number was not obtained in some cases because PCT staff members were not accustomed to ask caregivers for this information. These results indicate that a more rigorous monitoring in the beginning of the implementation period may be necessary to ensure a proper implementation of an electronic caregiver record. PCT staff expressed ethical concerns about establishing medical records for caregivers with no formal disease diagnosis. Formal documentation of supportive and preventive initiatives for caregivers seemed to represent a new way of thinking in the interdisciplinary work. However, towards the end of the implementation period, the procedure seemed to have been accepted by PCT staff and fidelity increased. It became clear that the electronic records enabled correspondence with professionals in the health care system, particularly the general practitioner and psychiatric services. Documentation of caregiver assessments and support has been sparsely described in the literature. One survey examining bereavement practices among ten UK hospices in 2007 found that the practice of documenting information about caregivers varied. Some hospices documented caregiver information in the patient’s record, and only a minority of hospices had introduced separate caregiver records due to data protection legislation issues [13]. However, the study did not address the feasibility of creating caregiver records. Overall, documentation in separate caregiver records seems to be a novel task for palliative care staff.

### Strengths and limitations

To the best of our knowledge, this study is the first to test the feasibility of an intervention based on the “*Bereavement support standards*”. This study was performed according to recognised guidelines for the process evaluation of complex interventions, which secures the necessary systematic approach and transparency. Using this method has helped us identify barriers to the implementation of systematic risk and needs assessment, which may be taken into consideration in future studies. Moreover, to our knowledge, this study is the first to evaluate the feasibility of the establishment of a separate and formal medical record for caregivers. The electronic records for caregivers may be an important tool to promote documentation, systematic planning of support, follow-up, quality assessment and information sharing between professionals.

Some limitations should also be addressed. The *risk and needs assessment form* has not been validated. However, this study shows that the form was considered helpful by professionals in clinical practice. Furthermore, because the “*Bereavement support standards”* are complex and comprehensive, the intervention in this study was based on selected key elements. Hence, the feasibility of applying the entire set of standards is still to be tested. Assessment of caregiver outcomes and caregivers’ response to the intervention was beyond the scope of this study and studies are needed to determine the potential effect of the standards on caregiver distress during caregiving and bereavement.

Regarding the sample of caregivers, selection bias may constitute a limitation. The sample of participating caregivers was only 46% of eligible caregivers, and it was not possible to assess if this was a representative sample. Still, all eligible caregivers present at the first home visit, were asked to participate consecutively over the course of ten months. Hence, the population is estimated to be fairly representative of caregivers in specialised palliative care in Denmark.

Finally, we adapted the “*Bereavement support standards*” to the specific context of a palliative home care team in a European setting. The results from this study may therefore not apply to all settings. Still, the barriers to implementation identified in our study seem comparable to barriers found in Australian and British studies, which indicates that the results are likely to be relevant to other palliative care settings.

## Conclusion

This study showed that an intervention based on key elements of the “*Bereavement support standards*” was feasible. PCT staff reported that the intervention was acceptable and useful, and that their effort to support caregivers early in the caregiving trajectory intensified as a result of the intervention. Still, several barriers were identified in the initial phase of the implementation, i.e. the point of view that the ad hoc assessment was sufficient, a shortage of time at the first home visit to conduct the assessment, and ethical considerations about establishing medical records for caregivers with no formal diagnosis. These barriers should be taken into consideration in future studies on the application of bereavement support standards in palliative settings. Systematic assessment of caregivers’ risk and support needs has the potential to ensure that support in palliative care reaches the caregivers who need it. Future studies are needed to evaluate the intervention on caregiver level, test if the intervention results in a more precise identification of caregivers at risk and how the intervention may affect caregivers’ bereavement outcomes.

## Abbreviations

PCT: palliative care team
MRC: Medical Research Council
CSNAT: caregiver support needs assessment tool.
